# The role of immunotherapy prior to liver transplantation in advanced or down-staged HCC – timing of transplants, selection of patients, and hurdles to overcome with immunosuppression

**DOI:** 10.1097/JS9.0000000000003520

**Published:** 2025-11-04

**Authors:** Parissa Tabrizian, Albert Chan, Rebecca Marino, Héloïse Giudicelli, Allan Lam, Manon Allaire

**Affiliations:** a Liver Transplant and Hepatobiliary Surgery, Recanati/Miller Transplantation Institute, Icahn School of Medicine at Mount Sinai, One Gustave L. Levy, New York, NY, USA; b Liver Transplant Center, Queen Mary Hospital, Department of Surgery, The University of Hong Kong, Hong Kong, China; c AP-HP Sorbonne Université, Hôpital Universitaire Pitié-Salpêtrière, Service d’Hépato-gastroentérologie, Paris, France; d AP-HP Sorbonne Université, Hôpital Universitaire Pitié-Salpêtrière, Service d’Hépato-gastroentérologie, Paris, France; INSERM UMR 1138, Centre de recherche des Cordeliers, Paris, France

**Keywords:** Bridging, Downstaging, Hepatocellular carcinoma, Immunosuppression, Immune-related adverse events, Immune checkpoint inhibitors, Liver transplantation, Neoadjuvant immunotherapy

## Abstract

Immune checkpoint inhibitors (ICIs) have revolutionized the management of hepatocellular carcinoma (HCC). Landmark trials, including IMbrave150 and HIMALAYA, have established combination ICI therapies as the standard of care for advanced HCC. Beyond these indications, ICIs are now being explored as neoadjuvant therapies for liver transplantation (LT), offering potential benefits in downstaging tumors and serving as bridging therapies for patients outside traditional transplant eligibility criteria. While early evidence from case reports and trials highlights promising oncologic and survival outcomes, the current understanding of ICI use before LT introduces new, unexplored challenges. Allograft rejection, safety concerns regarding immune-related adverse events, and the effective management of the complex interplay between immunosuppression and immune therapy represent ongoing themes of discussion. The tumor microenvironment and immunomolecular profiles, which critically influence ICI efficacy, remain the true cornerstones of patient stratification that are yet to be fully understood. Additionally, the lack of reliable biomarkers and the limitations of current radiologic criteria complicate treatment planning and response evaluation. Overcoming these obstacles requires multidisciplinary collaboration, refined patient selection strategies, and robust clinical trials to establish the safety and efficacy of ICIs in this context. This review aims to provide an up-to-date summary of the ever-evolving role of ICIs in LT strategies, offering a comprehensive overview of proposed therapeutic protocols, their oncologic and safety profiles, and the integration of emerging biological insights into clinical practice.

## Introduction

Over the past decade, immune checkpoint inhibitors (ICIs) targeting PD-1, PD-L1, and CTLA-4 have become key treatments for many solid cancers, including hepatocellular carcinoma (HCC). The IMbrave 150 and HIMALAYA trials established the superiority of ICI combinations, such as Atezolizumab with Bevacizumab and Durvalumab with Tremelimumab, for advanced-stage HCC, setting a new standard of care^[1,2]^.

With proven efficacy in advanced stage HCC, there is growing interest in exploring ICIs as neoadjuvant therapy for liver transplantation (LT) in patients outside traditional eligibility criteria^[3,4]^. The primary focus has been on their potential as a neoadjuvant regimen, raising the question of whether they can serve as an effective downstaging option or act as a “bridge” to transplantation.

The rationale for neoadjuvant immunotherapy in preoperative management of HCC encompasses several distinct objectives. Neoadjuvant therapy can be used to downstage tumors, converting locally advanced or advanced HCC into transplantable disease by reducing tumor burden to within eligibility criteria. It may also enable conversion to surgery by shrinking previously unresectable tumors to allow for hepatic resection. In the setting of LT, bridging therapy refers to treatments for patients already listed for LT, aimed at preventing tumor progression and minimizing the risk of waitlist dropout. In addition to maintaining transplant eligibility, bridging therapy may also help reduce post-transplant recurrence by addressing micrometastatic disease.

Early data come primarily from case reports and small studies with heterogenous cohorts.^[5–31]^ Recent evidence from a prospective multicenter study shows promising results, with a 3-year survival of 85% and 76%, after LT, of patients successfully downstaged to within Milan Criteria (MC)[27]. Data from ongoing trials like EMERALD-1 and LEAP 012 (NCT04246177) support the efficacy of ICIs in both advanced and intermediate stages of HCC, suggesting potential benefits in both bridging therapy and downstaging in patients awaiting LT^[32,33]^.

However, the use of ICIs in the LT setting remains in its early stages (Table 1 reported experience on ICI pre-LT), with concerns about allograft rejection, optimal patient selection, and safety issues such as treatment-related adverse events^[5–33]^. While the potential for ICIs in LT is significant, especially for bridging or downstaging, the impact on graft function and oncologic outcomes is not yet well understood.

**Table 1:** Summary of ICIs as neoadjuvant strategy in liver transplant

Author	Year	Number of Patients	Underlying Liver Disease	Milan Criteria	Pre-ICI tumor size	Macrovascular Involvement	ICI	ICI Duration	ICI as bridge to transplant?	ICI as first downstaging option?	Downstaged?	Other Pre-transplant Treatments	Time Interval between last ICI and LT	Rejection	Recurrence	Death within 12 months
Nordness. et al[5]	2020	1	HCV	Yes	NR	No	Nivolumab	2 years	Yes	No	Yes	Resection, Radioembolization, TACE, Sorafenib	8 days	Acute rejection, 6 POD	N/A	Yes
Schwacha-Eipper et al[6]	2020	1	Alcoholic Cirrhosis	No	1 (2.5 cm) 3 (< 2 cm)	No	Nivolumab	34 cycles	No	No	Yes	Resection, Sorafenib, Regorafenib, Microwave ablation	21 weeks	No	No	No
Chen et al[7]	2021	1	HBV	Yes	N/A	No	Toripalimab, Lenvatinib	10 cycles, unknown	No	No	No	Resection, TACA, Sorafenib, MWA, RFA	93 days	Acute Hepatic Necrosis, 1 POD	N/A	Yes
Chen et al[8]	2021	5	Cirrhosis of unknown etiology	No	NR	No	Nivolumab	1 cycle (3), 6 cycles (2)	No	No	NR	TACE, Resection, 3 RFA	59–122 days	No	2 Recurrences (metastatic disease)	Unknown
Dehghan et al[9]	2021	1	HCV	Yes	2 (2.5 cm; 1.0 cm)	No	Nivolumab	16 cycles	No	No	Yes	TACE, MWA, Sorafenib	5 weeks	Subacute Hepatic Necrosis (10 POD), Graft loss, Re-transplant successful	Unknown	Unknown
Lizaola-Mayo et al[10]	2021	1	NASH	Yes	1 (2.8 cm)	No	Nivolumab, Ipilimumab	6 months	No	No	Yes	Radioembolization, Sorafenib	8 weeks	No	No	No
Qiao et al[11]	2021	7	Unknown	NR	NR	NR	Pembrolizumab or Camrelizumab + Lenvatinib	1–5 cycles	Yes	NR	Yes	No	40 days	Acute cellular rejection (10 POD)	Unknown	Unknown
Sogbe et al[12]	2021	1	HBV	No	13 (largest 47 mm)	No	Durvalumab	15 months	No	No	Yes	Resection, Sorafenib	90 days	No	No	No
Tabrizian et al[13]	2021	9	5 HBV; 2 HCV; 1 NASH; 1 None	6 Yes 3 No	NR	NR	Nivolumab	2–32 cycles	Yes	No	NR	Chemo- and Radioembolization, Ablation, Radiation	4 weeks	1 mild rejection due to low Tacrolimus levels	No	No
Schnickel et al[14]	2022	5	4 HCV; 1 HBV	Unknown	NR	No	Nivolumab	8–18 months	NR	NR	NR	No	10 days—83 months	1 Acute hepatic necrosis (14 POD), Graft loss, Re-transplant successful	No	No
Aby et al[15]	2022	1	HCV	No	3 (2.0 cm, 2.4 cm 2.4 cm)	Yes; PVT	Nivolumab	23 cycles	No (intended as destination therapy)	No	Yes	Chemo- and Radioembolization, MWA, Sorafenib	16 days	Acute cellular rejection (9 POD)	Unknown	No
Abdelrahim et al[16]	2022	1	HCV	Yes	1 (5 cm)	No	Atezolizumab, Bevacizumab	6 cycles 5 cycles	No	Yes	No (new 8 mm lesion but shrink of main lesion 3.3 cm)	No	2 months	No	No	No
Yin et al[17]	2022	1	HBV	No	NR	No	Lenvatinib + PD-1 inhibitor	6 months	No	No	NR	Resection, MWA, TACE	NR	Yes	NR	NR
Kang et al[18]	2022	1	NR	Yes	NR	No	Pembrolizumab	3 cycles	Yes	No	No	Cisplatin, Doxorubicin, Dexrazoxane, TACE, Resection	138 days	No	No	No
Dave et al[19]	2022	8	4 HCV; 1 HBV; 1 NASH; 2 Other	Yes (7)	NR	NR	Nivolumab	Unknown	Yes	NR	NR	Mean of 2 Loco-regional treatments	105 days	2 rejections; Graft loss, Re-transplant successful	Unknown	1 Yes
Wang et al[20]	2023	16	14 HBV; 2 ALD	No	range 1.5–10 cm	3	2 Nivolumab 7 Pembrolizumab. 4 Sintilimab 2 Camrelizumab 1 Multiple	1–27 cycles	Yes	NR	Yes	Yes	1–184 days	9 Acute liver rejection	5 Yes	Unknown
Rudolph et al[21]	2023	1	Unknown	Unknown	NR	No	Nivolumab	7 cycles	NR	No	NR	Resection, Bland Embolization, SIRT, MWA	55 days	GVHD (35 POD)	No	No
Chouik et al[22]	2023	1	Alcoholic Cirrhosis	No	6 cm	No	Atezolizumab, Bevacizumab	18 cycles	No (intended as destination therapy)	NA	Yes	No	1 week	No	No	No
Ohm et al[23]	2023	3	2 HCV	2 No	range 2.2–3.75 cm	No	2 Atezolizumab + Bevacizumab 1 Ipilimumab + Nivolumab	6-7 cycles	No	Yes	2 No	SBRT, Y-90	2-7 days	No	No	No
			1 HBV	1 Yes							1 Yes					
Giudicelli et al[24]	2023	1	NAFLD	No	10 cm	Yes; PVT	Atezolizumab, Bevacizumab	9 cycles	No	Yes	Yes	Resection, radioembolization	6 months	No	No	NR
Xu et al[25]	2024	25	19 HBV; 6 Other	19 No	NR	NR	17 PD-1 inhibitors	2–10 cycles	No	No	NR	TKI, TACE, Resection, Ablation, Radiation, Oncolytic Virus	40–151 days	3	12	4
							6 ICI combinations									
				5 Yes												
Abdelrahim et al[26]	2024	6	2 NASH 4 Viral	4 No	range 3–4.8 cm	Yes; PVT	4 Atezolizumab + Bevacizumab 1 Nivolumab + Ipilimumab 1 Nivolumab	1–42 cycles	No	NR	Yes	TACE, Y-90, SBRT, RFA	5 months	No	1	5 No
				2 Yes												1 Yes
Tabrizian et al[27]	2024	117	56 HCV	31 Yes	range 2.9–7.2 cm	2 Yes	68 Nivolumab	4–16 cycles	No	No	65 Yes	TACE, Y-90, Ablation, Other	13–120 days	7	5	4
			23 HBV													
							24 Atezolizumab + Bevacizumab									
			17 NASH													
			14 ALD													
							21 Pembrolizumab									
				86 No												
			7 Other													
							4 Durvalumab + Tremelimumab									
			3 None													
Guo et al[28]	2024	83	63 HBV	27 Yes	range 3.5–10 cm	12 Yes; PVT	31 Camrelizumab	2–6 cycles	15 Yes	No	Yes	TKI, TACE, Resection	NR	23 Yes	20 Yes	5 Yes
							18 Pembrolizuamb									
			4 HCV													
							14 Sintilimab									
			16 Other													
				56 No												
							11 Tislelizumab									
							4 Nivolumab									
							5 Atezolizumab									
Liu et al[29]	2024	9	1 Alcoholic Cirrhosis	9 No	range 2.1–19.6	3 Yes	5 Atezolizumab + Bevacizumab	NR	No	No	4 Yes	TACE, MWA, Y-90	3 months	1	No	NR
			2 MASH													
			4 HCV													
							1 Ipilimumab + Nivolumab									
			1 PSC				1 Nivolumab									
							1 Pembrolizumab									
			1 Other													
							1 Combination									
Kumar et al[30]	2024	1	HBV	No	12.6 cm	Yes; PVT	Atezolizumab, Bevacizumab	6 cycles	No	Yes	Yes	SBRT	6 weeks	No	No	No
Tabrizian et al[31]	2024	17	6 HCV	16 Yes	range 6–12.1 cm	No	Atezolizumab + Bevacizumab	5–18 cycles	No	1 Yes	13 Yes	TACE, Resection, MWA, Y-90	41–123 days	2	1	2
			4 HBV													
			3 MAFLD													
			1 Alcoholic Cirrhosis	1 No												
			3 Other													
				

**Abbreviations**: GVHD (Graft Versus Host Disease); MWA (Microwave Ablation); NR (Not Reported); NA (Not Applicable); PVT (Portal Vein Thrombus); RFA (Radiofrequency Ablation); SIRT (Selective Internal Radiation Therapy); TACE (Trans-Arterial ChemoEmbolization)

This review aims to explore the current understanding of ICI use in HCC downtaging before LT, addressing key challenges like allograft rejection, safety concerns, and the complex interaction between immunosuppression and immune therapy.

This article is compliant with the TITAN Guidelines 2025 governing the declaration and use of AI in scientific research[34].

## Guiding principles of liver immunology in transplantation

The liver’s anti-inflammatory environment is shaped by a complex network of immune cells – including Kupffer cells (KCs), which have both pro- and anti-inflammatory functions, regulatory T cells and myeloid-derived suppressor cells (MDSCs). This diverse cellular landscape creates a tolerogenic niche, supporting immune tolerance in the healthy liver but also creating a tumor-friendly microenvironment that allows immune evasion and promotes hepatocarcinogenesis. In non-tumor liver tissue, a balance between effector cells (CD8 + T cells, NK cells, dendritic cells) and regulatory populations (Tregs, MDSCs, KCs) maintains immune homeostasis. In contrast, the HCC tumor microenvironment is characterized by increased infiltration of immunosuppressive cells, functional exhaustion of CD8 + T cells, and a shift toward pro-tumorigenic macrophages[35].

While this immunosuppressive environment supports tumor growth, it also explains the renewed effectiveness of ICIs. However, the diverse nature of the HCC microenvironment leads to varying responses to ICI therapy^[35,36]^.

HCC is classified into two categories based on its immunomolecular profile: inflamed and non-inflamed. Inflamed HCC, accounting for about 30% of cases, has abundant immune cells, high expression of immune checkpoints (PD-1/PD-L1), overactive Interferon (IFN) signaling, and fewer chromosomal alterations. It is further divided into immune-active, immune-like (linked to CTNNB1 mutations), and immune-exhausted subclasses^[37,38]^. Inflamed HCC is more likely to respond to ICIs and has better oncologic outcomes. In contrast, non-inflamed tumors, often associated with TP53 mutations and limited immune activity, generally have a poorer prognosis and lower response to immunotherapy[38].

The rationale for using ICIs in HCC lies in the interaction between immune checkpoint ligands and receptors, which regulate immune cell function to prevent tissue damage. Activating checkpoints like CD28 promote T cell expansion, while inhibiting checkpoints (e.g., PD-1, CTLA-4, LAG3, and TIM3) suppress it^[39,40]^.

Current immunotherapy strategies focus on enhancing antitumor immunity with ICIs, monoclonal antibodies that block inhibitory checkpoints. Though several gene signatures have been linked to favorable ICI responses, none are validated for clinical use, complicating patient selection[40].

**HIGHLIGHTS**

- Immune checkpoint inhibitors (ICIs) are redefining hepatocellular carcinoma (HCC) care, with groundbreaking trials IMbrave150 and HIMALAYA setting a new therapeutic standard for advanced disease.
- The emerging role of ICIs as neoadjuvant therapies for HCC before liver transplantation (LT) offers potential benefits in downstaging tumors and bridging patients outside traditional eligibility criteria.
- The risk of post-transplant rejection following ICI therapy may be reduced by an adequate washout period and increased immunosuppression post-LT; plasmapheresis has been reported in select cases of severe or steroid-refractory rejection.
- Complete or partial response to ICIs, alone or with locoregional therapy, does not eliminate recurrence risk in HCC. Integrated assessment of tumor biology and response characteristics is essential to define transplant candidacy.
- Challenges in using ICIs before LT remain managing allograft rejection, balancing the complex interplay of immunosuppression and immune activation, and addressing safety concerns related to immune-related adverse events.
- This review provides a comprehensive overview of current evidence, ongoing trials, and strategies to guide the clinical application of neoadjuvant ICIs in the pre-LT setting for HCC. It emphasizes the importance of multidisciplinary collaboration and the need for prospective clinical trials.

ICIs targeting PD-1 and CTLA-4 restore CD8 + T cell function and activation. However, they can provoke allograft rejection by disrupting the liver’s tolerogenic environment, creating a challenge in combining ICIs with immunosuppressive therapies critical for transplantation. While immunosuppression prevents allogeneic immune responses post-LT, ICIs enhance immune responses to cancer cells^[36,40–42]^.

### Mechanism of action of ICIs

The PD-1/PD-L1 interaction plays a key role in maintaining graft tolerance post-LT, inducing T-cell exhaustion to suppress immune responses. However, in the HCC tumor microenvironment, this interaction facilitates immune evasion and tumor survival. Inhibiting PD-1/PD-L1 restores CD8 + T cell function, enhancing immune responses against the tumor^[40,43]^.

CTLA-4, an inhibitory receptor on T cells, regulates immune responses by preventing T cell activation. In HCC, upregulation of CTLA-4 contributes to immune evasion. Blocking CTLA-4 with antibodies like Ipilimumab and Tremelimumab reactivates T cells, boosting anti-tumor immunity by increasing CD4 + and CD8 + T cell activity^[40,42]^.

The rationale behind combining PD-1/PD-L1 inhibitors with VEGF inhibitors is to enhance immune responses through complementary mechanisms[44]. VEGF inhibition reduces immune evasion by decreasing MDSCs and Tregs, while controlling the PD-1/PD-L1 axis boosts CD8 + T cell, dendritic cell, and NK cell activation. VEGF inhibitors also normalize blood vessels, improving T cell infiltration and dendritic cell maturation, which enhances the overall immune response. This combination offers a synergistic effect, improving the response to ICIs in inflamed HCC and overcoming limitations in non-inflamed tumors^[44,45]^.

## Advanced stage HCC: the rationale of ICI as a downstaging strategy

The introduction of ICIs has presented a new option for the management of advanced HCC. Phase I and Phase II trials have proven the efficacy of ICI monotherapy, leading to further investigation of combination therapy in two landmark trials, the IMBRAVE 150 trial and the HIMALAYA trial^[1,2]^. These trials demonstrated improved overall survival using combination ICI therapy compared to sorafenib, and established combination ICI therapy as the standard of care for advanced HCC that are not amendable for locoregional therapy (LRT) or surgery. These results were further confirmed by the CheckMate-9DW study, which showed that first-line treatment with nivolumab and ipilimumab improved overall survival compared to the investigators’ TKI of choice (sorafenib or lenvatinib)[46].

Following these promising results, studies have been conducted to explore the use of ICIs in neoadjuvant and downstaging therapy for both resectable and initially unresectable HCC. For neoadjuvant therapy, early studies have reported major pathological response (≥70% necrosis) in 20-33% of patients who had received neoadjuvant immunotherapy followed by surgical resection of HCC^[12,22,27]^. A prospective study of 64 HCC patients treated with ICIs before LT showed promising results, with 78% achieving downstaging to within Milan criteria, and 37% proceeding to transplant. The study found that pre-transplant ICI therapy led to a median downstaging time of 7.2 months, compared to 3-4 months for patients receiving standard LRTs. While survival outcomes after transplant were similar across groups, the study suggests that preoperative immunotherapy may improve downstaging and outcomes for high-risk patients, though larger studies are needed to confirm its effectiveness as a bridge to transplantation[27].

For initially unresectable or non-transplantable HCC, successful outcomes, such as downstaging in patients with portal vein thrombosis, radiological response with cumulative objective response rate by modified RECIST criteria of 34% (95% CI, 30.3–37.8; I^2^ = 60.3), and varying conversion-to-resection rates from 15.9% to 80% have been reported^[47–49]^. However, there is currently no robust guideline regarding ICIs as a downstaging treatment for HCC before surgery or LT.

### Current experience in combining LRT with ICI

There is also significant interest in combining ICIs with LRT such as transarterial chemoembolization (TACE), transarterial radioembolization (TARE), and radiotherapy[39]. The rationale is that these LRTs induce cellular damage and tumor necrosis, leading to release of tumor neoantigens, with subsequent synergistic activation of the immune system and the complementary anti-tumor response[50]. The EMERALD-1 trial demonstrated that the combination of ICIs and TACE is superior to TACE alone for objective responses and progression-free survival (hazard ratio [HR] 0.77, 95% CI 0.61–0.98) for TACE-eligible unresectable HCC[32]. The LEAP 012 trial (NCT04246177) a phase 3, multicenter randomized, double blind study investigated the combination of lenvatinib (an oral tyrosine kinase inhibitor) with pembrolizumab (anti PD1 monoclonal antibody) in combination with TACE. Early results show superior Progression-Free Survival (PFS) of 14.6 months with the combination therapy compared to 10 mins for those receiving placebo and TACE alone with a 34% reduction in the risk of disease progression or death (HR 0.55, *P* = 0.0002). Evidence for ICI combined with TARE is limited, though a retrospective study suggest improved survival comparing with ICI only (adjusted HR 0.50, 95% CI 0.36-0.68)[51]. In the START-FIT trial, Chiang et al suggested a novel sequel approach of trimodality therapy, with TACE and stereotactic body radiotherapy (SBRT) followed by ICI administration. It was reported that 42% of patients with locally advanced HCC attained radiologic complete response, with 55% of patients successfully downstaged to be eligible for curative treatment[52]. A phase 2, single arm study demonstrated that combining (selective internal radiation therapy) SIRT with nivolumab is a feasible treatment for advanced HCC, achieving an ORR of 41.5%, a median time to progression of 8.8 months, and a median OS of 20.9 months, with an acceptable safety profile[53]. These findings indicate that ICI in combination with LRT offers a more robust and effective treatment regimen than ICI only.

Table 2 shows a summary of ongoing trials combing LRT with ICIs.

**Table 2:** Summary of ongoing trials involving ICIs and LRT. Search terms “HCC AND ICI” were used on the clinicaltrials.gov website. The results were subsequently screened for studies involving combination therapy with locoregional therapy (LRT). All ongoing trials were included, except those that did not specify the type of LRT used. Trials incorporating additional treatments, such as tyrosine kinase inhibitors (TKIs), were also included. The data source was clinicaltrials.gov

NCT Number	Acronym	LRT	Pharmaceutical Agent	Stage of HCC
NCT06710223		Cryoablation + Hepatic Artery Infusion of SD-101	Durvalumab + Tremelimumab	Advanced
NCT06375317		HAIC	Adebrelimab + Regorafenib	Advanced
NCT05886465			Atezolizumab + Bevacizumab	Advanced
NCT05233358			ICI + Regorafenib	Advanced
NCT06632106			ICI + TKI	Advanced
NCT05573282			ICIs + Regorafenib	Advanced
NCT06041477	HAIC		PD-1 inhibitors + Lenvatinib	Intermediate + Advanced
NCT05166239			PD-1 inhibitors + Lenvatinib	Advanced
NCT05713994	CCGLC-001		PD-1 Inhibitors + TKI	Advanced
NCT05582278		HAIC/DEB-TACE	Tislelizumab + Lenvatinib	Advanced
NCT05992220	ALERT-HCC	External Beam Radiotherapy	Atezolizumab + Bevacizumab	Advanced
NCT04430452		Hypofractionated Radiotherapy	Durvalumab + Tremelimumab	Advanced
NCT06389422			Pembrolizumab	Advanced
NCT05339581	iPLENTY-pvtt	Intensity Modulated Radiotherapy	PD-1 Inhibitors	Advanced
NCT05396937		Multifocal Stereotactic Radiotherapy	Atezolizumab + Bevacizumab	Advanced
NCT06339424	PhotonAB	Photon Radiotherapy	Atezolizumab + Bevacizumab	Advanced
NCT05625893		Proton Radiotherapy	Atezolizumab + Bevacizumab	Advanced
NCT06133062	ProtonAB		Atezolizumab + Bevacizumab	Advanced
NCT06725121	MacroTrans	Radiotherapy	Atezolizumab + Bevacizumab	Advanced
NCT03316872			Pembrolizumab	Advanced
NCT04727307	AB-LATE02	RFA	Atezolizumab + Bevacizumab	Early
		RFA		
NCT05277675	RANT		Tislelizumab/Sintilimab + Lenvatinib/Bevacizumab	Recurrent
NCT05803928		RFA/MWA	PD-1 Inhibitors + Lenvatinib	Early, Recurrent
NCT06248554		MWA	PD-1 Inhibitors	Early
NCT06538935		SBRT	Adbelimumab + Apatinib	Not Specified
NCT05488522			Atezolizumab + Bevacizumab	Advanced
NCT04857684			Atezolizumab + Bevacizumab	Early + Intermediate
NCT05096715			Atezolizumab + Bevacizumab	Advanced
NCT06040177			Cardonilizumab + Lenvatinib	Advanced
NCT04913480			Durvalumab	Advanced
NCT05286320			Pembrolizumab + Lenvatinib	Advanced
NCT06313190	HSBRT2402		Sintilimab	Early
NCT05776875		TACE	Atezolizumab + Bevacizumab	Intermediate
NCT04712643			Atezolizumab + Bevacizumab	Advanced
NCT04224636	DEMAND		Atezolizumab + Bevacizumab	Advanced
NCT06024252	CHANCE023		Atezolizumab + Bevacizumab	Advanced
NCT03937830		TACE	Durvalumab + Bevacizumab + Tremelimumab	Advanced
NCT05301842	EMERALD-3		Durvalumab + Tremelimumab ± Lenvatinib	Advanced
NCT06487663	TIKET		ICI	Advanced
NCT05717738	CCGLC-008		ICI + TKI/ Anti-VEGF	Advanced
NCT04472767			Ipilimumab/Nivolumab + Carbozantinib	Advanced
NCT04268888	TACE-3		Nivolumab	Intermediate
NCT04340193	CheckMate 74 W		Nivolumab + Ipilimumab/Nivolumab	Intermediate
NCT04997850			PD-1 Inhibitors + Lenvatinib	Advanced
NCT05332821	CHANCE2201		PD-1/PD-L1 inhibitors + VEGF-TKI/bevacizumab	Advanced
NCT05332496	CHANCE2202		PD-1/PD-L1 inhibitors + VEGF-TKI/bevacizumab	Intermediate
NCT03397654	PETAL		Pembrolizumab	Not specified
NCT04246177			Pembrolizumab + Lenvatinib	Advanced
NCT03143270		DEB-TACE	Nivolumab	Advanced
NCT04229355			PD-1 Inhibitors	Advanced
NCT04814030	AIPD-1	TACE + HAIC	PD-1 Inhibitors	Advanced
NCT04814043	PLATIC		PD-1 inhibitors + Lenvatinib	Early + Intermediate
NCT05984511		TACE + I-125 Seeds Brachytherapy	Atezolizumab + Bevacizumab	Advanced
NCT04988945		TACE + SBRT	Durvalumab + Tremelimumab	Advanced
NCT05751343		TACE + HAIC	Atezolizumab + Bevacizumab	Advanced
NCT06323382		TACE/ HAIC	PD-1 Inhibitor + Bevacizumab	Advanced
NCT06669377		TACE + 125 Iodine Irradiation Stent	ICI + molecular targeted therapy	Advanced
NCT03259867	TATE-PD1	TATE (Trans-arterial Tirapazamine Embolization)	Nivolumab	Advanced
NCT05620771		Y90 Radioembolization	Atezolizumab + Bevacizumab	Advanced
NCT05377034	STRATUM		Atezolizumab + Bevacizumab	Advanced
NCT03099564			Pembrolizumab	Advanced
NCT03033446			Nivolumab	Advanced
NCT04605731			Durvalumab + Tremelimumab	Advanced
NCT03380130	NASIR-HCC		Nivolumab	Advanced

### ICI as a “Bridging” strategy to transplant

LT provides a radical treatment approach by removing the primary tumo and reducing the risk of intrahepatic recurrence associated with cirrhosis. However, limited availability of liver graft makes it inaccessible as an immediate treatment option for most patients^[4,54]^. Prolonged waiting time potentially leads to waitlist dropout, as disease progression may render patients ineligible for transplantation. To address this, bridging therapies are recommended to slow disease progression in patients with prolonged waiting periods for LT. The latest ESOT consensus report recommends bridging therapy for all eligible patients, citing evidence of improved long-term post-transplant survival and 5-year survival rates exceeding 80% in those who received bridging therapy[55]. While these results are primarily based on LRT, ICIs are now being explored, especially in patients with high-risk features or limited response to standard treatments. A recent case series analyzed nine patients who received nivolumab as bridging therapy prior to LT, with eight of nine patients having their final dose within 1 month of surgery. All nine successfully underwent transplantation, and at a median follow-up of 16 months, there were no cases of severe allograft rejection, tumor recurrence, or death. Explant pathology showed greater than 90% tumor necrosis in three patients, highlighting the potential effectiveness of ICI bridging for tumor control[13]. In a separate report, Chen et al described five patients beyond MC who were treated with nivolumab as bridging or downstaging therapy, with a mean interval of 64 days between the last ICI dose and transplant[7]. While none developed biopsy-proven acute rejection post-transplant, two had recurrent HCC. In contrast, Nordness et al reported a fatal case in a patient who received nivolumab for nearly 2 years, with the last dose administered eight days before transplantation; despite achieving complete radiological and pathological response, the patient developed fulminant hepatic necrosis and graft failure from severe rejection, resulting in death within 10 days post-transplant[5].

The use of ICIs may provide effective disease control, treat micrometastatic disease and help reducing waitlist dropout. It could also offer an *in vivo* assessment of sensitivity to ICIs by evaluating radiological and pathological responses^[4,13,30,39]^. This approach provides valuable prognostic information, as complete pathologic response has been shown to be associated with improved recurrence-free and overall survival post LT. Hence, using ICI as bridging therapy may also potentially guide adjuvant therapy decision after LT.

### Treatment response and transplant candidacy

Recent multicenter studies underscore the complexity of transplant decision-making in HCC patients who achieve a complete or partial response (PR) to ICI-based regimens, with or without LRT^[56,57]^. In the largest international cohort to date, complete radiological response (CR) to ICI occurred in 4.4% of patients but conferred exceptional survival, with 1- and 3-year OS rates of 98% and 86%, respectively, and a median RFS exceeding 30 months. However, approximately one third of CR patients experienced recurrence during follow-up, indicating that radiological CR does not exclude residual disease, and questioning whether such patients should receive automatic transplant priority. Notably, 89% of those who underwent resection or transplantation after ICI CR had complete pathological response on explant, while most patients who remained under surveillance had survival outcomes similar to surgical cohorts, supporting an individualized, biology-driven approach to LT candidacy[56]. Chiang et al further demonstrate that, when combining LRT with immunotherapy for initially unresectable HCC, CR rates may reach 46%, but recurrence remains common (34.5% in CR patients), though salvage therapies are often feasible. Their analysis found that smaller tumor burden and absence of macrovascular invasion were key predictors of CR, reinforcing the need for multidisciplinary risk stratification in transplant selection[57].

In both cohorts, marked Alpha-Fetoprotein (AFP) declines frequently accompanied radiological responses. Scheiner et al found AFP dropped below 10 ng/ml in 84% of CR patients with initially elevated levels, suggesting that morphologically stable disease with substantial AFP reduction may indicate improved prognosis, although robust data for prioritizing LT solely on AFP response are lacking. Both studies emphasize that neither radiological nor biomarker response alone should dictate transplant candidacy^[56,57]^. Selection should be guided by integrated assessment of response durability, tumor biology, AFP trends, and patient comorbidities.

## Immune-related adverse events and their management

### Which toxicities? With which molecule?

While immunotherapy offers significant benefits in various cancer management, it can also lead to several complications, such as immune-related adverse events (irAEs), which result from an overstimulated immune response^[58–60]^. The type and frequency of irAEs vary depending on the specific class of ICIs (Table 3). Anti-PD-(L)1 inhibitors induce preferably skin disorders, endocrine abnormalities (such as hypothyroidism, hyperthyroidism, and hypophysitis), and pulmonary issues, particularly pneumonitis. Anti-CTLA-4 inhibitors are more commonly linked to gastrointestinal complications (enterocolitis), liver toxicity (hepatitis), and endocrine disorders (hypophysitis). When these two classes are combined, the risk of irAEs, especially severe ones, increases, with gastrointestinal, hepatic, and endocrine toxicities becoming the most prevalent[59]. Skin toxicity occurs in over 50% of patients, manifesting as maculopapular rashes, lichenoid reactions, psoriasis, or bullous dermatoses. Endocrine toxicity, particularly hypothyroidism, is also frequent, often requiring lifelong hormone replacement therapy. Gastrointestinal toxicity, including diarrhea and abdominal pain, can progress to severe colitis or even intestinal perforation in extreme cases. Although most irAEs are manageable with appropriate interventions, some can be life-threatening. Myocarditis, while rare, may lead to heart failure or death; pneumonitis can cause acute respiratory failure; hepatitis can progress to acute liver failure; and neurological toxicities such as myasthenia gravis, Guillain-Barré syndrome, and encephalitis may potentially cause paralysis or respiratory failure. The rarest irAEs are the most severe and lethal^[60,61]^. In the positive phase III trial for advanced HCC[1], the incidence of irAEs was consistent with the rates reported in the literature for all types of cancers, as summarized in Table 3.

**Table 3:** **Immune-related adverse events according to the type of ICIs and in Phase 3 HCC trials**^[54,55]^

	Dermatologic	Gastrointestinal	Hepatic	Endocrine	Pulmonary	Cardiovascular	Neurological	Grade 3/4
All cancer	Rash (10-15%) Pruritus (10-15%) Stevens-Johnson syndrome or toxic epidermal necrolysis (<1%)	Diarrhea (35%) Colitis (5-10%) Intestinal perforation (<1%)	Hepatitis (10-20%) Severe hepatitis (1-5%)	Hypothyroidism (5-10%) Adrenal insufficiency (1-2%) Hypophysitis (3%)	Pneumonitis (2-5%)	Myocarditis Pericarditis Arrhythmias (rare <1% but severe)	Encephalitis Meningitis (rare but severe)	50-70%
All cancer-Anti CTLA4	All dermatological iRAEs (50%)	Diarrhea (35%) Colitis (12%)	Hepatitis (1-25%)	Endocrinopathies (10%) Hypophysitis (4,5%) Hypothyroidism (6%)	Pneumonitis (<1%)	- All cardiovascular iRAEs (<1%)	- All neurological iRAEs (3,8%)	34%
All Cancer-Anti PD1	All dermatological iRAEs (50%)	Diarrhea (20%) Colitis (1%)	Hepatitis (1-6%)	Endocrinopathies (4-14%) Hypophysitis (<1%) Hypothyroidism (8-14%)	Pneumonitis (1-4%)	- All cardiovascular iRAEs (<1%)	All neurological iRAEs (6%)	10%
All Cancer-Anti PDL1	All dermatological iRAEs (50%)		Hepatitis (1-6%)	Hypophysitis (<1%)	Pneumonitis (2-3%)	- All cardiovascular iRAEs (<1%)	All neurological iRAEs (1-5%)	12%
HCC IMBRAVE 150 (atezolizumab-bevacizumab)	Rash (10%) Prurit (14%)	Diarrhea (11%)	Hepatitis (14%)	Hypothyroidism (10%)	Pneumonitis (1%)			43%
HCC HIMALAYA (tremelimumab-Durvalumab)	Rash (22%) Prurit (23%)	Diarrhea (26%)	Hepatitis (10%)	Hypothyroidism (12%)	Pneumonitis (1%)	Myocarditis (0,3%)		51%
HCC CHECKMATE 9DW (Nivolumab-ipilimumab)	Rash (6%) Prurit (8%)	Diarrhea (4%)	Hepatitis (6%)	Hypothyroidism (3%)				41%
HCC CARES-310 (Camrelizumab-rivoceranib)	Rash (4%) RCCEP (30%)	Diarrhea (4,5%)	Hepatitis (1%)	Hypothyroidism (13%)				81%

RCCEP = reactive cutaneous capillary endothelial proliferation

### Management of irAEs

Managing irAEs requires a multidisciplinary approach, with close monitoring, prompt diagnosis, and rapid intervention being key to achieving optimal outcomes. Collaboration between oncologists, immunologists, and other specialists is critical, as irAEs can affect multiple organ systems and require specialized input. In some centers, multidisciplinary meetings focused specifically on irAEs management have been established to facilitate this teamwork. Decisions regarding the discontinuation of immunotherapy should always be made collectively, considering the risks of irAEs recurrence, cancer progression, and patient safety.

The administration of corticosteroids or other immunosuppressive agents is often necessary to manage irAEs, but this must be balanced against the risk of infection and the potential cancer progression, as shown in Table 4[60]. Corticosteroids should be administered at the lowest effective dose for the shortest possible duration to minimize side effects, such as immunosuppression and metabolic complications. Abrupt cessation of corticosteroids must be avoided due to the risk of adrenal insufficiency and other severe complications, making gradual tapering essential.

**Table 4:** **Management of immune-related adverse events**[56]

	Organ supply specific measures	Infection prophylaxis	First Line	Second line	Third line	Fourth line
Colitis	Fluid resuscitation Antidiarrheal agents Nutritional support Discuss endoscopic evaluation	Antifungals Antivirals Pneumocystis jirovecii prophylaxis	Corticosteroids IV (methylprednisolone 1-2 mg/kg/day to 3-4 mg/kg/day) Response should be closely monitored if no improvement within 48-72 hours, escalation of therapy is warranted	Anti TNF-α (infliximab)	Vedolizumab	
Hepatitis	Avoid hepatotoxic agents			Mycophenolate Mofetil Anti IL-6 (tocilizumab)	If severe: Tacrolimus	If severe: Azathioprine or Cyclosporine
Uveitis				Anti TNF-α (infliximab)	Mycophenolate Mofetil	
Encephalitis	If severe, plasmapheresis or IV immunoglobulins			Mycophenolate Mofetil Azathioprine Methotrexate	If severe: Rituximab (anti-CD20) Tacrolimus	If severe: Cyclophosphamide
Haematological symptoms	Growth factor support If severe, plasmapheresis or IV immunoglobulins			Rituximab (anti-CD20)	Mycophenolate Mofetil	If severe: Cyclosporine Anti IL-6 R
Severe bullous skin disease	Consider burn unit care Topic corticosteroids			Rituximab (anti-CD20) or Anti TNF-α (infliximab) or Anti IL-6 (tocilizumab)	If severe: Tacrolimus	If severe: Cyclosporine
Interstitial pneumonitis	If severe, high-flow nasal cannula or mechanical ventilation Empiric antibiotics IV immunoglobulins			Anti IL-6 (tocilizumab) or Anti TNF-α (infliximab)	Mycophenolate Mofetil	If severe: Cyclophosphamide
Myocarditis	If severe intensive monitoring and support (inotropes) cardiologist consultation			Anti IL-6 (tocilizumab) or Mycophenolate Mofetil or	Abatacept (anti-CD80/6)	If severe: Alemtuzumab (anti-CD52)

Nephritis	Avoid nephrotoxic agents Hydratation Discuss renal replacement therapy					
Pancreatitis	Hydratation, pain control					
Rheumatological symptoms	Nonsteroidal anti-inflammatory drug			Hydroxychloroquine, Sulfasalazine or Methotrexate	If severe: Anti IL-6 R	If severe: Anti TNF-α
Endocrinopathies	Hormone replacement therapy		For painful thyroiditis or hypophysitis, corticosteroids (prednisolone or methylprednisolone 0,5 to 1 mg/kg/day)			

For patients with irAEs that are refractory to corticosteroids or for severe cases, additional immunosuppressive agents may be required. These options include:

- Biologic Disease-Modifying Anti-Rheumatic Drugs (bDMARDs): Agents such as TNF-α inhibitors, gut-specific immunosuppressants, and anti-CD20 monoclonal antibodies are often used for moderate to severe irAEs that do not respond adequately to corticosteroids.
- Conventional Synthetic DMARDs (csDMARDs): Drugs like methotrexate, azathioprine, and mycophenolate mofetil can be employed in specific cases, particularly when long-term immunosuppression is needed.
- Targeted Synthetic DMARDs (tsDMARDs): Medications like Janus kinase (JAK) inhibitors may be used when more targeted immunosuppression is indicated, especially in cases where conventional options fail.
- Other Immunomodulators: Intravenous immunoglobulins (IVIg) can be considered in rare or particularly severe irAEs, providing another immunosuppressive option when conventional therapies are insufficient.

The choice of immunosuppressive therapy should be highly individualized, considering the type of irAE, its severity, the patient’s underlying malignancy, and their overall health status. Personalizing the approach to immunosuppression helps to maximize efficacy while minimizing risks.

An emerging strategy in irAE management involves targeting specific cytokines with cytokine inhibitors. These agents can offer advantages over corticosteroids, including a potentially shorter duration of symptoms and effectiveness in steroid-refractory cases. However, the impact of cytokine inhibitors on anti-tumor immunity remains a concern, as these agents may interfere with the body’s ability to fight cancer. Additional challenges, such as cost and accessibility, must also be considered. Despite these limitations, cytokine inhibitors hold promise as a more refined, effective method of managing irAEs, particularly for patients with difficult-to-treat cases.

## Acute cellular rejection (ACR): definition, diagnosis, and optimal washout period

### Definition and diagnosis

Immunosuppressive treatment and immunotherapy share common targets that make the situation of immunotherapy utilization before LT complex. Indeed, immunotherapy will delete inhibitory signals which are usually triggered after antigen recognition and may lead to graft rejection.

After LT, acute cellular rejection (ACR) can cause graft rejection in 10-25% of LT recipient[62]. ACR is caused by the recognition by recipient T-cells of non-self-donor alloantigens on antigen presenting cells (APCs)[63]. Antigen recognition results in proliferation and activation of T-cells in lymphoid tissue before migration to the allograft. During the alloimmune response, T cells activation requires three signals. The first signal is involving T-cell receptor triggered by donor antigen on the surface of APCs. The second signal or “costimulation” signal is not antigen-specific and involves some pairs of surface molecules of T-lymphocytes and APCs like B7/CD28 and CD40/CD40L pathways. These two signals activation results in numerous factors production, including interleukin-2 (IL-2), CD25 and CD40 ligand. The third signal is due to IL-2 binding to its high-affinity receptor (that contains CD25), followed by activation of the mTOR (“target of rapamycin”) pathway and results in T-cell clonal proliferation and effector T-cells generation[64]. Diagnosis of ACR is histological. Liver biopsy shows T cells and other leukocytes infiltration, evidence of ductular injury and endothelitis. Severity of rejection is graded with Histological Banff Classification (Table 5)[65]. ACR is considered to require treatment when Banff score is over 6. ACR usually improves with steroids pulses and/or with escalation of immunosuppression[66].

**Table 5:** Banff Score for diagnosis of acute cellular rejection adapted from ^[65]^

Category	Criteria	Score
**Portal inflammation**	Mostly lymphocytic inflammation involving, but not noticeably expanding, a minority of the triads.	**1**
	Expansion of most of all the triads, by a mixed infiltrate containing lymphocytes with occasional blasts, neutrophils and eosinophils.	**2**
	If eosinophils are conspicuous and accompanied by edema and microvascular endothelial cell hypertrophy is prominent, acute antibody mediated rejection should be considered.	
	Marked expansion of most or all the triads by a mixed infiltrate containing numerous blasts and eosinophils with inflammatory spillover into the periportal parenchyma.	**3**
**Bile duct inflammation damage**	A minority of the ducts are cuffed and infiltrated by inflammatory cells and show only mild reactive changes such as increased nuclear: cytoplasmic ratio of epithelial cells.	**1**
	Most or all of the ducts infiltrated by inflammatory cells. More than an occasional duct shows degenerative changes such as nuclear pleomorphism, disordered polarity and cytoplasmic vacuolization of the epithelium.	**2**
	As above for 2, with most or all the ducts showing degenerative changes of focal luminal disruption.	**3**
**Venous endothelial inflammation**	Subendothelial lymphocytic infiltration involving some, but not a majority of the portal and/or hepatic venules.	**1**
	Subendothelial infiltration involving most or all of the portal and/or hepatic venules or without confluent hepatocyte necrosis/drop out involving a minority of perivenular regions.	**2**
	As above for 2, with moderate or severe perivenular inflammation that extends into the perivenular parenchyma and is associated with perivenular hepatocyte necrosis involving a majority of perivenular regions.	**3**

### Optimal washout period

The optimal washout period between the last dose of ICI and LT remains debated. Current guidelines suggest a washout of 8-12 weeks based on case series, which studies showing a higher risk of rejection in patients with shorter interval^[5–31]^. For instance, Dave et al found that acute rejection occurred in patients with washout period under 90 days[19], while Wang et al noted median washout of 21 days in patients who experienced rejection compared to 60 days in those who did not[20].

A systematic review and meta-analysis of 91 patients showed a 26.4% rejection rate, mostly cellular, in patients treated with ICIs prior to LT[3] while a U.S. prospective multicenter study reported a 16.7% rejection rate in 43 transplanted patients previously treated with ICIs with a median time to rejection of 14 days[27]. Importantly, most cases of graft rejection were successfully managed with medical therapy and did not significantly impact overall survival[27]. However, key factors such as a shorter interval between the last ICIs dose and LT, as well as younger patient age, are associated with higher rejection risks. In a recent international retrospective cohort of 119 HCC patients receiving ICIs before LT, a washout period >50 days reduced rejection rates to levels comparable with non–ICI-exposed patients, without adversely affecting recurrence-free survival[67]. While ICIs hold potential as a pre-transplant strategy, further large-scale, prospective studies are needed to refine protocols and identify which patients are most likely to benefit from this approach while minimizing the risk of graft rejection[68].

In deceased donor LT, time constraints regarding the optimal washout period create uncertainty, highlighting the need for prompt and precise coordination between the transplant and hepatology-oncology teams. In contrast, living donor transplantation can bypass these logistical concerns, allowing for more tailored coordination between ICI administration and the timing of the transplant[4]. LDLT might provide a unique opportunity to optimize the timing of preoperative ICIs, potentially enhancing the immunotherapeutic response while minimizing the risk of irAEs, as the scheduling of surgery is more predictable and can be adjusted based on the patient’s clinical response. However, potential drawbacks include the risk of immune-related complications in the donor liver and limited data on the long-term impact of preoperative ICIs on graft survival and recipient outcomes, necessitating extreme caution, particularly in LDLT^[4,69]^.

## Patient selection

There are currently no official guidelines or consensus on the ideal profile of HCC patients who should receive neoadjuvant ICI regimens. The decision to administer immunotherapy, either alone or with LRT is guided by several different guidelines including the AASLD, EASL, and ILTS ^[70–72]^. Intention to treat studies including Vitality and trials including EMERALD 1 and LEAP 012 have shown that immunotherapy can effectively downstage tumors^[27,32]^. Emerging data from the ongoing ImmunoXXL study (NCT05879328) and MERITS-LT consortium are helping define potential candidates[33]. The ImmunoXXL study is showing promising results with the atezolizumab-bevacizumab combination, demonstrating that patients with tumor beyond up to seven criteria (excluding EHS and Vp4) who achieved sustained downstaging, had excellent survival outcomes. Similarly, MERITS-LT consortium proposes a flowchart for managing LT candidates with HCC beyond MC, recommending immunotherapy for patients with residual or recurrent disease after LRT and a required 12-week washout period before LT^[73,74]^.

**Figure 1.**
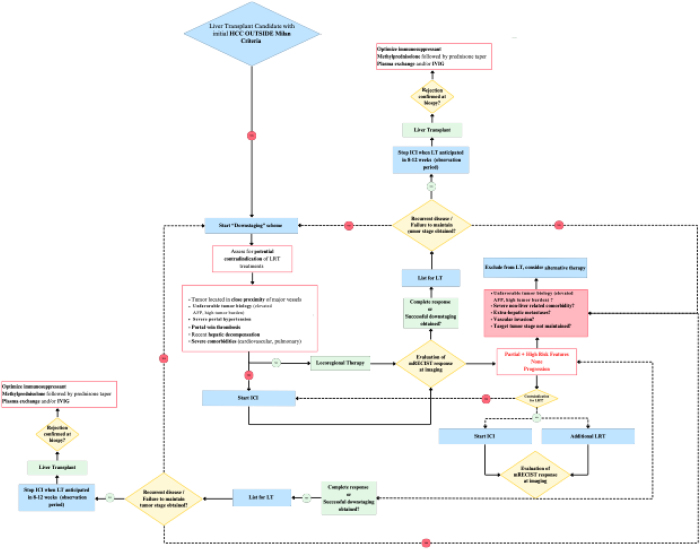
Comprehensive management algorithm to guide patient’s selection in LT setting. LT, liver transplantation.

Despite these advances, identifying the right candidates for preoperative immunotherapy remains challenging. The key is accurately assessing tumor response to optimize patient selection, though the lack of reliable biomarkers and difficulties interpreting radiological responses complicate this process. Additionally, pre-treatment and on-treatment biopsies are limited by the heterogeneity of HCC and the risks associated with invasive sampling, highlighting the need for better methods to assess response and stratify patients. A proposed algorithm to guide patients’ selection is represented in Figure 1.

## Basic concept of post-LT immunosuppression: finding the right balance

The fundamental concept of post-LT immunosuppression is finding a delicate balance between preventing graft rejection and preserving the patient’s immunity against infections and malignant tumors. Current immunosuppressive regimens, which typically include corticosteroids, calcineurin inhibitors, and mycophenolate mofetil, primarily target the adaptive immune response to suppress the activation of alloreactive T and B cells. However, this immunosuppression can hinder the immune system’s ability to control residual tumor cells or micrometastases, thereby increasing the risk of HCC recurrence. On the other hand, reducing immunosuppression to enhance antitumor immune surveillance may elevate the risk of graft rejection. Therefore, the challenge is to develop personalized immunosuppressive strategies that consider both the individual risk of rejection and the need for an effective antitumor immune response[68].

### Decrease the risk of ACR

There are many factors unique to the liver that may diminish ACR risk compared to other transplanted organs:

- MHC class II antigens are less strongly expressed in the liver (compared with the kidney and heart for example).
- The hepatic immune system is highly regulated to avoid inappropriate responses to antigens in the portal blood related to benign gut flora.
- Transplant surgery induces blood loss with some degree of wash-out of circulating immunotherapy agents.
- Massive immunosuppression is begun upon reperfusion of the liver, shutting down T-cell responses[4].

But, since immunosuppression goals are lowered in HCC recipients to limit tumor recurrence after LT, it is potentially exposing these patients and especially those previously treated with immunotherapy to a higher risk of ACR due to the activation of the immune response^[5,16]^.

The immunological tolerance of the graft is facilitated by the PD-1 and CTLA-4 pathways. PD-L1 is expressed in post-LT allografts, and PD-1 is substantially expressed on graft- infiltrating T-cells. Conversely, CTLA-4 binding to its counter-receptor B7 on T-cells produces an inhibitory signal that stops T-cell responses. As a result, blocking these pathways may cause these T-cells to become more active, resulting in T-cell-mediated graft rejection. This effect can be mitigated by an adequate washout period, increased immunosuppression post-LT, as well as the use of plasmapheresis[13].

- Age and immunotherapy washout length seem to be related to the ACR risk, and a 3-months washout may reduce it to that of patients without immunotherapy pre-LT exposure.

- Concerning post-LT immunosuppression management, it usually includes a combination of high-dose corticosteroids, calcineurin inhibitors and mycophenolate mofetil. Induction of immunosuppression with T cell-depleting agents, such as anti-thymocyte globulin, induces a profound depletion of T cells and might be an effective way to avoid immunotherapy-related ACR; however, lymphodepletion is known to induce surviving lymphocytes to undergo proliferation and differentiation into memory T cells that may impair the development of graft tolerance in the long run[33].

To date, there is insufficient evidence to propose a specific immunosuppressive regimen in these patients treated with immunotherapy before LT[16] but death-censored rejection-free survival is reportedly higher in patients receiving at least one drug in addition to corticosteroids.

Moreover, there is a clear lack of data from large-scale clinical trials showing superior outcomes in patients who had immunotherapy prior to LT compared to the standard-of-care and large prospective studies are necessary to confirm these results^[3,13,16,24]^.

### Decrease the risk of post-LT HCC recurrence

HCC recurrence occurs in approximately 5% to 20% of liver transplanted patients. This recurrence typically manifests within the initial years post-LT, is aggressive, and mostly extra-hepatic, and remains the leading cause of death after LT, with a median survival of 2 years post-LT^[75–77]^.

As the immune system plays a crucial role in modulating malignancy development, immunosuppressive drugs may negatively impact this malignancy development risk.

Mechanistic target of rapamycin inhibitors (mTORi) have unique antiproliferative effects that may reduce HCC recurrence. Unfortunately, the level of evidence remains limited with conflicting results among studies. In a recent meta-analysis involving three randomized controlled trials and four prospective cohort studies, no difference was observed between the two groups (512 patients treated with everolimus versus 494 patients treated with calcineurin inhibitors (CNIs) regarding HCC recurrence (RR: 1.22, 95% CI: 0.66-2.29, *P* = 0.52) and mortality (RR: 0.85, 95% CI: 0.48-1.50, *P* = 0.57). Then, it is not recommended to initiate immunosuppressive therapy with mTOR inhibitors to limit HCC recurrence[78].

Conversely, it has been raised that anticalcineurin inhibitors (CNIs), corticosteroids, mycophenolate mofetil, and azathioprine may increase the risk of HCC recurrence after LT but data remain too limited to extend any strong recommendation and most recently, in a large study performed on 22,535 LT patients, the induction immunosuppression was not associated with worse post-LT outcomes in patients transplanted with HCC exception priority[79].

Currently, consensus recommendations on the optimal management of immunosuppression in HCC recipients are inconsistent. The guidelines of the American Association for the Study of Liver Diseases (AASLD) and the European Association for the Study of Liver Diseases (EASL) do not address induction therapy in this context^[69,70]^. Conversely, the International Liver Transplantation Society (ILTS) and the Società Italiana Trapianti D’Organo guidelines highlight the importance of CNIs and overall immunosuppression minimization in HCC recipients, although they do not provide guidance on whether this should be accomplished with the assistance of induction therapy^[80,81]^.

## AASLD, EASL, and ILTS-ILCA recommendations

The AASLD, EASL, and ILTS-ILCA guidelines offer a unified message of caution regarding the use of ICIs in LT settings for HCC^[69–71]^. Their recommendations are based primarily on case series and single-institution retrospective studies, with a notable absence of level-one evidence to support routine clinical application of ICIs, despite promising exploratory outcomes. All three guidelines advise against the use of ICIs following LT, citing the substantial risk of acute graft rejection and advocating for TKIs as the preferred systemic option in cases of recurrence not amenable to surgical or LRTs. For patients awaiting transplantation, LRTs such as TACE, TARE, and ablation remain the recommended modalities for bridging or downstaging across all guidelines, with immunotherapy considered investigational and its use best confined to prospective clinical trials or highly selected scenarios discussed by multidisciplinary teams.

AASLD distinguishes itself by recommending a minimum 3-month discontinuation of ICIs prior to transplantation to mitigate the risk of graft rejection, a specific interval not endorsed by either EASL or ILTS-ILCA[69]. The latter two guidelines, call for individualized decision-making due to insufficient evidence defining a safe washout period^[70,71]^. Additionally, ILTS-ILCA uniquely addresses the growing interest in combining ICIs with LRTs or TKIs for downstaging, but maintains that LRTs alone should remain standard practice until further data become available – a position echoed by both AASLD and EASL, who also discourage combined strategies outside research settings. Overall, while all three guidelines align on risk minimization and the experimental status of ICIs in LT, they differ on specifics such as the ICI discontinuation timeline and the use of combination approaches^[69–71]^. Current guidelines remain cautious, pending results from prospective and randomized clinical trials.

## Ongoing challenges

The challenges in applying neoadjuvant ICIs for HCC revolve around four keys issues: the lack of standardized pathologic endpoints, reliance on RECIST criteria, unclear optimal treatment duration, absence of reliable biomarkers for treatment selection, and lack of level one evidence data.

### Pathologic endpoints

The absence of validated pathological endpoints limits the evaluation of neoadjuvant ICIs in HCC. While metrics like pCR and MRP are used in other cancers, they many not fully reflect immunotherapy’s unique mechanisms. Current studies use varying thresholds for tumor necrosis (70% in cemiplimab and nivolumab trials, 90% in cabozantinib), highlighting the need for standardized, immunotherapy-specific benchmarks^[82–84]^.

### Radiological criteria

RECIST critera, which focus on tumor size, are inadequate for assessing neoadjuvant ICI responses. Immune-related effects like pseudoprogression can mask true responses. For example, patients in the cemiplimab trial showed pathologic response without meeting RECIST criteria, highlighting the limitations of size-based assessments.

### Treatment duration

The optimal duration of neoadjuvant ICI therapy is unclear, balancing efficacy with the risk of irAEs. The goal is to prime an immune response against micrometastases rather than solely eliminating the tumor. Shorter treatment regimens may prime the immune system while minimizing toxicities that could delay surgery^[4,16,27,54]^.

### Biomarkers

Despite increasing use ICIs in HCC, no clinically validated biomarkers reliably predict treatment response or resistance. While PD-L1 expression has shown predictive value in other cancers, in HCC both high and low PD-L1 tumors can respond to ICIs, limiting its utility in patient selection. Similarly, tumor mutational burden is generally low in HCC and does not correlate with response[85]. Recent research emphasizes the importance of the tumor immune microenvironment: tumors with “inflamed” profiles (high CD8 + T cell infiltration and interferon-γ gene signatures) are more likely to respond, whereas an immunosuppressive environment, or increased oncofetal gene expression (such as GPC3 and AFP), can drive primary resistance to ICIs. Single-cell and spatial transcriptomic analyses have revealed additional resistance mechanisms, such as the presence of exhausted or dysfunctional T cell populations, dense fibroblast stroma limiting immune infiltration, and immunosuppressive macrophage subsets^[37,38,85]^.

In terms of non-invasive markers, blood-based assays such as the CRAFITY score (combining AFP and CRP), circulating immune cell subsets – including increased neutrophil-to-lymphocyte ratio, reduced circulating CD8 + T cells, and altered proportions of regulatory T cells and myeloid-derived suppressor cells (MDSCs) – as well as soluble PD-L1 and inflammatory cytokines, are all under active investigation, though none are yet clinically actionable[85]. Ultimately, ongoing multi-omic and single-cell studies are expected to yield new predictive markers and clarify mechanisms of both primary and acquired resistance. Neodjuvant therapy offers a unique opportunity to analyze biomarkers in surgically resected or explanted liver tissue, allowing for more targeted immunotherapy strategies in HCC.

### Lack of level one evidence

There is a lack of level one evidence supporting the use of neoadjuvant ICI in LT for HCC, emphasizing the need for robust clinical trials. Most current studies are small, non-randomized, or have limited follow up, making it difficult to draw definitive conclusions about the efficacy and safety^[5–31]^. Well-designed RCT are essential to guide clinical practice.

## Conclusion

The use of ICIs in HCC is expanding beyond advanced stages into earlier, potentially curative settings^[4,36,39]^. Evidence supporting combined TACE and ICIs suggests their potential to improve tumor control, reduce waitlist dropout, and enhance post-LT outcomes in high-risk patients. Early data indicate that pre-LT ICIs are safe and effective in select patients, offering a strategy for downstaging advanced tumors or maintaining durable responses while awaiting transplantation^[4,27,36,39,54]^. Integration of new biological insights into the tumor microenvironment and gene signatures associated with ICI response is driving the evolution of patient selection criteria, enabling more tailored and effective treatment strategies^[3,37,38]^. Key challenges include refining safety protocols, determining optimal washout periods, and managing post-LT immunosuppression. Ongoing controlled trials and advancements in understanding the biology of response, patient stratification, and organ allocation will be essential for optimizing their use. These developments could significantly improve outcomes and expand therapeutic options.

**Figure 2.**
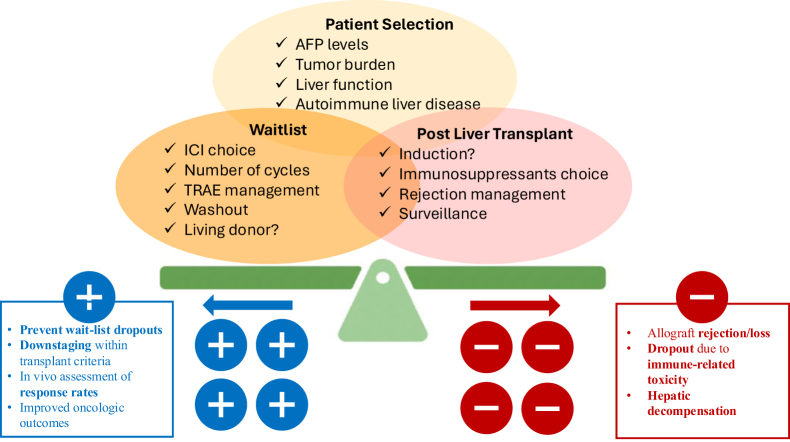
A summary of ongoing themes: striving to achieve the perfect balance among adequate patient selection, tailoring ICI treatments, and post-LT immunosuppression. ICI, Immune checkpoint inhibitor; LT, liver transplantation.

Figure 2 presents a summary of ongoing themes: aiming to achieve the optimal balance among appropriate patient selection, tailored ICI treatments, and post-LT immunosuppression.

## Data Availability

All data are available upon reasonable request from the corresponding author.
